# Carrot hairy roots: factories for secondary metabolite production

**DOI:** 10.1093/jxb/eraa435

**Published:** 2020-12-30

**Authors:** María A Pedreño, Lorena Almagro

**Affiliations:** Departamento de Biología Vegetal, Universidad de Murcia, Campus Universitario de Espinardo, Murcia, Spain

**Keywords:** Anthocyanins, antioxidant enzymes, black carrot, ethephon, hairy root, hydroxycinnamic acids, *Rhizobium rhizogenes*, *rol* genes

## Abstract

This article comments on:

**Barba-Espín G, Chen S-T, Agnolet S, Hegelund JN, Stanstrup J, Christensen JH, Müller R, Lütken H**. 2020. Ethephon-induced changes in antioxidants and phenolic compounds in anthocyanin-producing black carrot hairy root cultures. Journal of Experimental Botany **71**, 7030–7045.

**The use of hairy root (HR) cultures for the production of secondary metabolites is growing due to the high demand for bioactive food ingredients of plant origin that either have high nutritional value or act as powerful antioxidants, protecting the human body from oxidative processes generated by cellular metabolism.** Barba-Espín *et al.* (2020) **have developed an efficient protocol for establishment of anthocyanin-producing HR cultures from black carrots. In fact, fast-growing HR cultures obtained from root explants elicited with ethephon, an ethylene-releasing compound, substantially increased the anthocyanin content and the production of hydroxycinnamic acids. These results place these HR cultures as promising efficient systems to produce anthocyanins and phenolic compounds that have beneficial effects for human health.**

## Importance of secondary metabolites for human health and strategies to produce them

Plants are able to synthesize an enormous variety of compounds, particularly secondary metabolites. The precise functions of many of these compounds is largely unknown, but plants invest a large amount of energy into their synthesis because they contribute to plant survival. Many secondary metabolites are involved in defence reactions against different types of stress. However, the abundance of these compounds is often low (<1% DW) and their synthesis greatly depends on the plant genetic predisposition. Secondary metabolite production is often restricted to specific stages of plant development or the response to different stresses. Secondary compounds have caught the attention of the general public because most of them have beneficial effects on human health. Therefore, they are considered to be bioactive compounds. Thus, new strategies for the production of secondary metabolites have been developed (Thakur *et al.*, 2019; Almagro and Pedreño, 2020). Traditionally, these compounds can be directly obtained by extraction from plant raw material or by chemical synthesis, but these processes often have poor or inconsistent yields. In the first case, problems arise from the seasonal nature of plant growth, the heterogeneity of compounds, and, in some cases, the high risk of plant extinction, particularly if plant material is collected from the natural environment. In the case of chemical synthesis, stereo-specificity, the strict conditions of the biochemical reactions, and high costs of production are the main difficulties in the production of bioactive compounds. A biotechnological alternative for the production of secondary metabolites is the use of *in vitro* plant cultures, which have many advantages since they yield homogeneous extracts, they are grown in sterile conditions, and their maintenance is independent of seasonal or weather conditions. Therefore, HR cultures (see Box 1) have been developed as a promising strategy for metabolite production, especially in cases where the synthesis or extraction of bioactive compounds is difficult or unfeasible, or where harvesting plant material involves serious damage to the environment.

Box 1. **Hairy root technology: induction, culture, and bioactive compound production**

The transformation of root cultures with the naturally occurring soil-borne *Rhizobium rhizogenes* is a biotechnological method that is essentially independent of recombinant DNA techniques and, according to present legislation, it is not classified as a genetically modified organism. The presence of *R. rhizogenes* gives rise to the production of characteristic ‘hairy roots’ at the infection sites because of the insertion of a plasmid-borne transfer DNA (T-DNA), which (among other genes) contains the four *root oncogenic loci* (*rol*), termed *rolA*, *rolB*, *rolC*, and *rolD* (Mauro *et al.*, 2017). The axenic culture of HRs potentially offers numerous advantages: rapid and high-density growth in hormone-free media, genotypic stability, and the production of secondary metabolites at levels that are comparable with or even higher than those achieved in the wild-type plants.

The HR induction process usually involves cultivation of sterile wounded plant explants that are directly inoculated with an *R. rhizogenes* strain. Thereafter, explants are treated with antibiotics to eradicate the bacteria. The resultant HRs grow on hormone-free media. At this point, PCR is performed using primers that amplify *rol* and *vir* genes to conﬁrm that the roots are indeed HRs and not adventitious roots, and that the *R. rhizogenes* have been effectively eradicated. Once developed and selected, HR cultures must be maintained. Current procedures mainly use the maintenance method, which involves a monthly subculture of the HRs on solid and/or liquid culture media. The final step is the isolation and extraction of the required compounds.

One of the most used strategies involves genetically modified HRs that overexpress genes of interest that code for the key enzymes involved in complex metabolic pathways. Similarly, the use of elicitors is commonly used to induce the production of specific compounds that are naturally present in the plants, in the HR cultures. The selected compounds may be secreted into the media, facilitating easy and rapid downstream processing. However, some metabolites are retained within the cells, thus requiring a greater level of extraction and purification (Talano *et al.*, 2012). In such cases, strategies such as the permeabilization of the HR cells or treatment with organic solvents or surfactants is required to allow the release of the metabolite into the culture media (Chandra and Chandra, 2011). The metabolites expressed in the HRs can be highly specialized, and naturally produced by the plant or synthesized when metabolism is disturbed by the natural transformation with bacteria. Such systems can generate new compounds that are not produced by the untransformed roots (Hu and Du, 2006). In addition, new compounds could be produced by the insertion of specific genes that encode enzymatic steps of a given specific metabolic pathway (Guillon *et al.*, 2006).

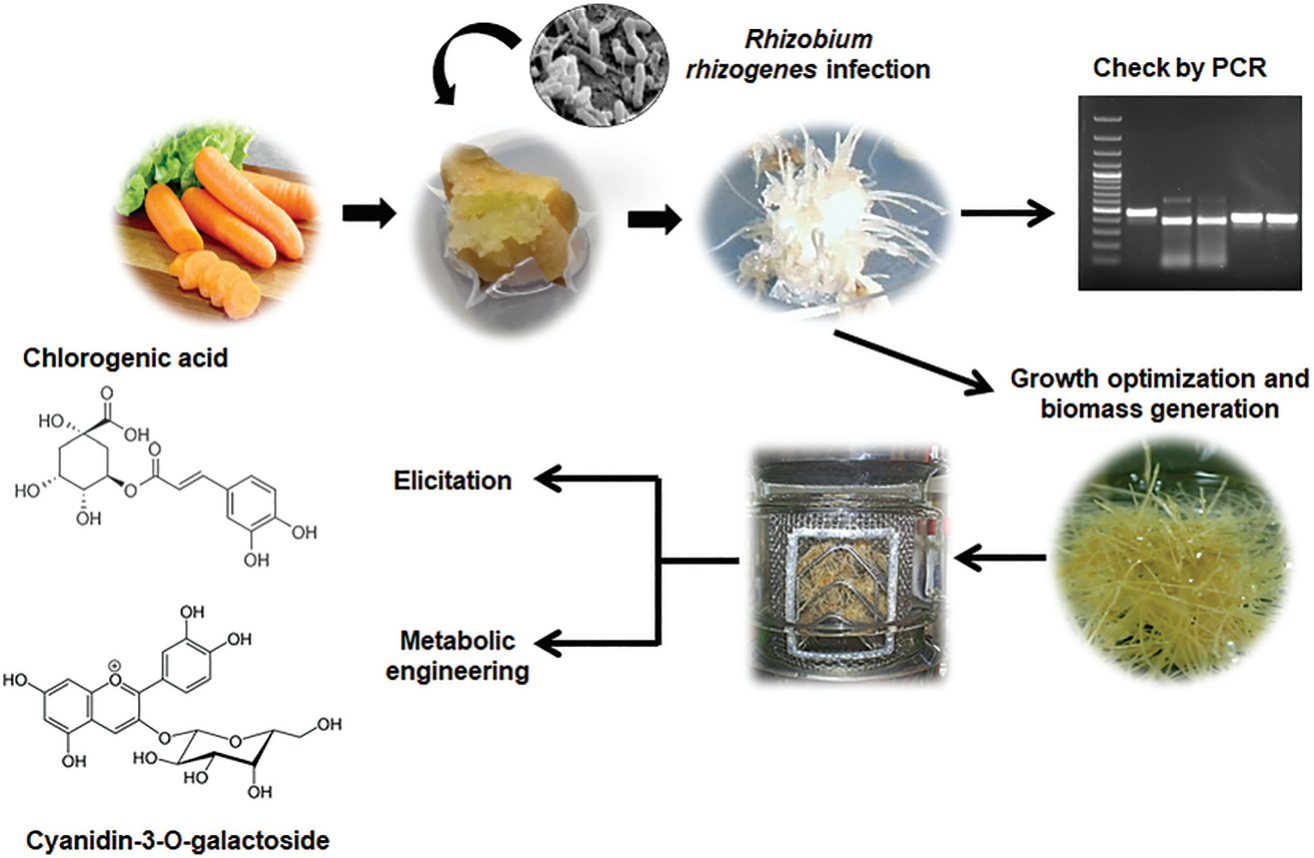

## Hairy roots as *in vitro* plant systems to produce secondary metabolites

HR cultures offer the advantage of continuous production over time as well as easy maintenance, rapid development, less cultivation time for biomass generation, and the capacity to simultaneously synthesize a wide variety of different bioactive compounds that can be used as ingredients for cosmetics, functional foods, preservatives, additives, and pharmaceuticals that are of commercial importance (Hu and Du, 2006). Due to their technological and economic advantages, the development of HR cultures has gained increasing interest by academic researchers, biotechnology companies, and pharmaceutical industries (Gutierrez-Valdes *et al.*, 2020). The development of optimal HR culture systems requires a consideration of the complexity of the compounds to be produced, together with the characteristics of the plant species selected for HR induction, because some species are more efficient than others in terms of productivity. This is particularly important with regard to the production of specialized metabolites, where the choice of the species and the bacterial infection strain used will influence the types of metabolites that will be produced (Gutierrez-Valdes *et al.*, 2020). Thus, in the study reported of Barba-Espín *et al.* (2020), black carrot (*Daucus carota* ssp. *sativus* var. *atrorubens* Alef.) was selected for HR induction by infection with a *Rhizobium rhizogenes* strain. These authors report the novel establishment of high anthocyanin-producing black carrot HR cultures. Over 90 viable HR lines were obtained; of these, three were selected that showed the most vigorous growth—named NB-R, 43-R, and 43-H. PCR analysis was performed using primers that amplify the presence of *rolB* and the absence of *vir* genes to conﬁrm that the roots were indeed HRs (and not adventitious roots) and that *R. rhizogenes* was efﬁciently eradicated (see Barba-Espín *et al.*, 2020). The presence of any *rol* genes, especially *rolB*, is crucial because they have been proven to be potent inducers of plant secondary metabolism. This strategy has been successfully used to increase the production of different bioactive compounds (Dilshad *et al.*, 2016, and references therein). In addition to the selection of highly compound-producing HR lines, a high biomass production capacity is necessary for selecting the most appropriate HR culture. It is necessary to optimize different physical parameters and nutritional requirements to achieve this goal (Chandra and Chandra, 2011). For this reason, Barba-Espín *et al.* (2020) grew the selected HR lines in liquid Murashige and Skoog (MS) medium at different strengths (1/4 MS, 1/2 MS, and full MS). The analysis of biomass accumulation and anthocyanin content was undertaken over a 4 week period. Surprisingly, there was not a clear separation in time between biomass accumulation and the accumulation of secondary metabolites. Although the use of 1/2 MS medium had a minor effect on biomass accumulation, it strongly stimulated anthocyanin production. This medium was therefore suitable for both biomass generation and anthocyanin accumulation.

The production of specialized metabolites in HR cultures, as in all plant-based production systems, usually requires an appropriate selection of elicitors; that is, substances which when applied at low concentrations can induce the synthesis of the desired compounds or increase their production in *in vitro* plant cultures. There is extensive information in the literature on the types of elicitor that can be used to produce specific metabolites in such *in vitro* plant culture systems (Gutierrez-Valdes *et al.*, 2020). Barba-Espín *et al.* (2020) chose to use ethephon, an ethylene-releasing compound, as an anthocyanin elicitor. Ethephon triggered a distinct response as a function of the time of application. When ethephon was applied at day 0, the elicitor decreased both biomass and anthocyanin contents, whereas when it was applied at day 10, the elicitor substantially increased anthocyanin accumulation with no detrimental effects on biomass production. This phenomenon can be explained by the less efficient enzymatic machinery for secondary metabolite production at the early phases of growth, resulting in a reduced elicitor response. The most pronounced difference between the elicited and control HRs was found in line 43-R at day 28, where an 82% increase in metabolite levels was observed following elicitation. Nine anthocyanins and 19 hydroxycinnamic acid derivatives were identified using UPLC-PDA-TOF during HR growth. Moreover, a high percentage of acylated anthocyanins (up to 78%) was observed in line 43-R. This finding highlights the potential applicability of black carrot HRs in colorant production, since these acylated anthocyanins show a greater stability in subsequent applications. Regarding phenolic compounds, 24 hydroxycinnamic acid derivatives were identified in the three HR lines. These contribute to anthocyanin stability in black carrots through intermolecular co-pigmentation reactions. The results reported by Barba-Espín and co-workers demonstrate the potential of HRs for the future biotechnological production of secondary metabolites and antioxidant compounds.

## Stepping forward

The use and application of HR cultures for the production of secondary metabolites is growing. However, some drawbacks still exist in terms of our current lack of knowledge on how to manipulate the system for the precise overexpression of complex metabolic pathways. The potential of genome editing and CRISPR/Cas9 technologies is starting to show some promising results in the field of HR cultures. These approaches provide promising alternatives for the production of important secondary metabolites, which will have a strong impact on the broad commercialization of HR cultures with several applications in the cosmetic, nutraceutical, and food industries.
